# Supplementary material for: Building functional connectivity neuromarkers of behavioral self-regulation across children with and without Autism Spectrum Disorder
Source: Dev Cogn Neurosci. 2019 Dec 5;41:100747. doi: 10.1016/j.dcn.2019.100747 (PMC6994646; doi:10.1016/j.dcn.2019.100747)
Supplement: Supplementary file 1 [file mmc1.pdf]

SUPPLEMENT

ASD > TD	GU Site	KK Site	Combined Sample	Split Half 1	Split Half 2
**Age**	0.045	-	-	-	-
**Males**	< 0.0001	-	0.004	-	0.022
**Motion (before)**	-	-	0.007	-	0.022
**Motion (after)**	< 0.0001	-	-	-	-
**SRS**	< 0.0001	< 0.0001	< 0.0001	< 0.0001	< 0.0001
**Inhibition**	< 0.0001	< 0.0001	< 0.0001	< 0.0001	< 0.0001
**Shifting**	< 0.0001	< 0.0001	< 0.0001	< 0.0001	< 0.0001
**Emotion Control**	< 0.0001	< 0.0001	< 0.0001	< 0.0001	< 0.0001
**TD > ASD**	**GU Site**	**KK Site**	**Combined Sample**	**Split Half 1**	**Split Half 2**
**FIQ**	-	< 0.0001	0.02	-	0.005
**VIQ**	-	0.001	0.031	-	0.006
**PIQ**	-	0.011	0.034	-	0.036

Supplementary Table S1. Significance of group differences between TD children and children with ASD. Scores for all three subscales of behavioral regulation were significantly higher in children ASD than for TD children in all analyzed samples, reflecting more issues with inhibition, shifting, and emotional control. We observed that in some, but not all samples, children with ASD had greater head motion and lower IQ. In addition, akin to the ratio in the general population, children with ASD tended to be predominantly male. Results are given as p-values of independent sample t-tests, which are adjusted in cases of unequal variance as assessed through Levene's test. Motion (in mm) refers to the absolute maximum displacement at any timepoint in the resting-state fMRI scan before and after motion mitigation and denoising procedures. SRS=Social Responsiveness Scale (total score); IQ=Intelligence Quotient; FIQ=full scale IQ; VIQ=verbal IQ; PIQ=performance IQ.
